# FXR agonists enhance the sensitivity of biliary tract cancer cells to cisplatin *via* SHP dependent inhibition of Bcl-xL expression

**DOI:** 10.18632/oncotarget.8964

**Published:** 2016-04-25

**Authors:** Wei Wang, Ming Zhan, Qi Li, Wei Chen, Huiling Chu, Qihong Huang, Zhaoyuan Hou, Mohan Man, Jian Wang

**Affiliations:** ^1^ Department of Biliary-Pancreatic Surgery, Ren Ji Hospital, School of Medicine, Shanghai Jiao Tong University, Shanghai, 200127, China; ^2^ Department of Biochemistry and Molecular Cell Biology, Shanghai Key Laboratory of Tumor Microenvironment and Inflammation, Institutes of Medical Sciences, School of Medicine, Shanghai Jiao Tong University, Shanghai, 200025, China; ^3^ The Wistar Institute, Philadelphia, PA 19104, USA

**Keywords:** farnesoid X receptor, cisplatin, small heterodimer partner, signal transducer and activators of transcription 3, biliary tract cancer

## Abstract

Chemoresistance is common in patients with biliary tract cancer (BTC) including gallbladder cancer (GBC) and cholangiocarcinoma (CC). Therefore, it is necessary to identify effective chemotherapeutic agents for BTC. In the present study, we for the first time tested the effect of farnesoid X receptor (FXR) agonists GW4064 and CDCA (chenodeoxycholic acid) in combination with cisplatin (CDDP) on increasing the chemosensitivity in BTC. Our results show that co-treatment of CDDP with FXR agonists remarkably enhance chemosensitivity of BTC cells. Mechanistically, we found that activation of FXR induced expression of small heterodimer partner (SHP), which in turn inhibited signal transducer and activator of transcription 3 (STAT3) phosphorylation and resulted in down-regulation of Bcl-xL expression in BTC cells, leading to increased susceptibility to CDDP. Moreover, the experiments on tumor-bearing mice showed that GW4064/CDDP co-treatment inhibited the tumor growth in vivo by up-regulating SHP expression and down-regulating STAT3 phosphorylation. These results suggest CDDP in combination with FXR agonists could be a potential new therapeutic strategy for BTC.

## INTRODUCTION

Biliary tract cancers (BTCs) are a heterogeneous group of tumors arising from the epithelial cells of the intra- and extra-hepatic bile ducts and gallbladder. Histologically, majority of BTCs are adenocarcinomas and have a poor prognosis. The majority of BTC patients exhibit an unresectable disease at the time of diagnosis due to the advanced cancer stage. Cisplatin (CDDP) is a first line chemotherapeutic drug to treat various types of human cancers including BTCs. However, CDDP resistance is common in patients with BTCs. Cytotoxicity of CDDP is mediated by its interaction with DNA and the formation of DNA adducts (mainly intrastrand crosslinks), thus activating several signal transduction pathways and culminating in the activation of apoptosis [1]. The combination of small molecular compounds with anticancer drugs aimed at bringing tumor cell populations into a state more susceptible to the cytotoxic effects of chemotherapeutic agents is a particularly interesting strategy in cancer chemotherapy [2, 3]. Therefore, in order to improve the therapeutic efficacy of CDDP to BTC, new agents that can enhance CDDP induced apoptosis need to be identified.

Farnesoid X receptor (FXR) is a well-characterized member of the so-called metabolic subfamily of nuclear receptors, and is a transcriptional sensor for bile acids [4, 5]. Significant progress has been made in the understanding of the role of FXR in carcinogenesis [6, 7]. However, the role of FXR in growth regulation, apoptosis, and cancer is still under evaluation, as separate studies have established both positive and negative correlations between FXR expression and cancer. The expression of FXR may vary depending on the site and type of the tumor. Liver and colon carcinomas, for instance, show a low FXR expression [8, 9], whereas some other tumors, such as esophageal and pancreatic carcinomas, show a high expression of FXR [10, 11]. Accordingly, activation of FXR results in a significant repression of cancer progression in liver and colon carcinomas [8, 12, 13], whereas inhibition of FXR suppress tumor progression in esophageal and pancreatic carcinomas [10, 11]. Our previous study has verified the reduced expression of FXR in human cholangiocarcinoma (CC) and gallbladder cancer (GBC) tissue. Moreover, administration of FXR agonist chenodesoxycholic acid (CDCA) and GW4064 resulted in a significant inhibition of tumor growth and induction of apoptosis in CC cell line QBC939 [14–16]. Based on previous studies, a possible explanation is that FXR activation induces small heterodimer partner (SHP) gene transcription which in turn induces apoptosis. Swales et al. reported that FXR is expressed in human breast cancer MCF-7 and MDA-MB-468 cells, and that activation of FXR by its ligands activates SHP gene transcription and induces cell apoptosis [17]. Growing evidence has demonstrated that SHP has a tumor suppressor function and is an active component of apoptosis signaling [18]. SHP activates apoptosis by translocating to mitochondria, binding to the anti-apoptotic protein Bcl-2, and disrupting Bcl-2/Bid interaction to cause cytochrome c release [19]. SHP also activates apoptosis by regulating miR-206 expression to block the anti-apoptotic activity of Notch3 [20]. The adamantyl-substituted retinoid-related (ARR) compounds AHPN and 3-Cl-AHPC bind directly to SHP, which promotes the formation of a corepressor complex containing Sin3A, nuclear receptor co-repressor (N-CoR) to activate apoptosis [21]. Therefore, activation of FXR-SHP axis to induce apoptosis might be promising therapeutic approach in treating BTC. In this study, we for the first time demonstrate that combination of FXR agonists and CDDP displays higher efficiency to induce apoptosis of human BTC cells via SHP dependent inhibition of STAT3 (signal transducer and activator of transcription 3) phosphorylation and then Bcl-xL expression.

## RESULTS

### FXR agonist enhances CDDP-induced inhibition of cell viability in BTC cells

**Figure 1:**
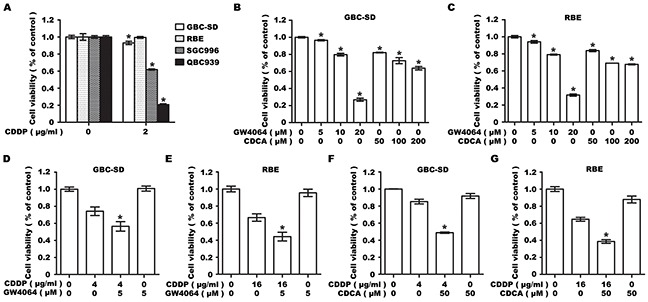
Farnesoid X receptor agonist GW4064 or CDCA enhances the sensitivity of GBC-SD and RBE cells to CDDP **A.** Cell viability in GBC-SD, SGC996, RBE and QBC939 cells treated with CDDP for 48h. Columns, mean of three experiments; bars, SD. *P <0.05, treatment group compared with control group. **B, C.** Cell viability in GBC-SD (B) and RBE (C) cells treated with GW4064 or CDCA for 48h. Columns, mean of three experiments; bars, SD. *P <0.05, treatment group compared with control group. **D, E.** Cell viability in GBC-SD (D) and RBE (E) cells treated with CDDP alone, GW4064 alone or CDDP/GW4064 co-treatment for 48 h. Columns, mean of three experiments; bars, SD. *P <0.05, combination treatment group compared with CDDP-alone group. **F, G.** Cell viability in GBC-SD (F) and RBE (G) cells treated with CDDP alone, CDCA alone or CDDP/CDCA co-treatment for 48 h. Columns, mean of three experiments; bars, SD. *P <0.05, combination treatment group compared with CDDP-alone group.

To examine the synergistic effect of FXR agonists and CDDP on cell viability, we first investigated the drug resistance in multiple human BTC cell lines, and chose two cell lines GBC-SD and RBE, showing significant resistance to CDDP (Figure 1A). The chosen cell lines were then analyzed for sensitivity to FXR agonists GW4064 and CDCA, and a dose-dependent inhibitory effect on cell viability was determined (Figure 1B, 1C). Low doses of GW4064 (5μM) and CDCA (50μM) were then chosen for a combined treatment with CDDP to treat GBC-SD and RBE cells. No obvious reduction in cell number was observed in GW4064 or CDCA treated group, while co-treatment with CDDP led to a significant reduction in cell viability at 48 h, compared to CDDP treatment only (Figure 1D, 1E, 1F, 1G).

### FXR agonist enhances CDDP-induced apoptosis of BTC cells

**Figure 2:**
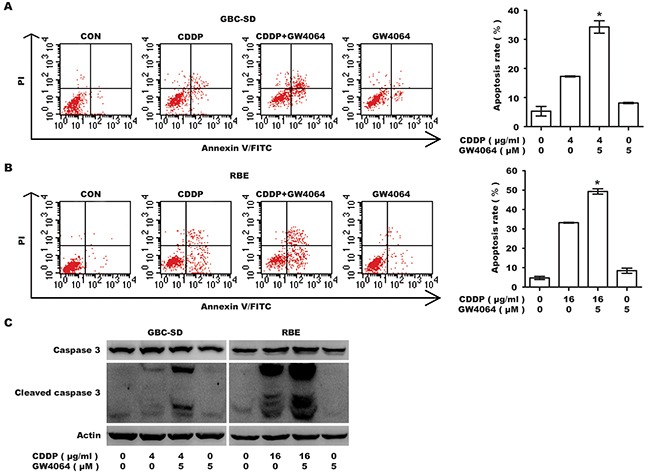
Farnesoid X receptor agonist GW4064 enhances the apoptosis induced by CDDP in GBC-SD and RBE cells **A, B.** Apoptosis rate analysis using Annexin V/PI flow cytometry in GBC-SD (A) and RBE (B) cells treated with CDDP alone, GW4064 alone and CDDP/GW4064 co-treatment for 48 h. Columns, mean of three experiments; bars, SD. *P <0.05, combination treatment group compared with CDDP-alone group. **C.** Level of total caspase 3 and cleaved caspase 3. Cells were exposed to CDDP alone, GW4064 alone and CDDP/GW4064 co-treatment for 36 h before harvested for IB.

To validate whether the repression in viability was attributed to an increase in apoptosis, Annexin V-FITC/PI double labeling flow cytometry was conducted. GW4064 markedly enhanced CDDP-induced apoptosis in GBC-SD cells (apoptosis rate from 17.28±0.14% to 34.27±1.51%) and RBE cells (apoptosis rate from 33.21±0.17% to 49.33±0.97%) (Figure 2A, 2B). In both cell lines, cleaved caspase 3 was significantly increased by GW4064/CDDP co-treatment, compared with CDDP alone (Figure 2C). Collectively, these data indicate apoptosis induced by CDDP is significantly enhanced by the co-treatment with FXR agonist GW4064.

### FXR agonist/CDDP co-treatment additively inhibits Bcl-xL expression

**Figure 3:**
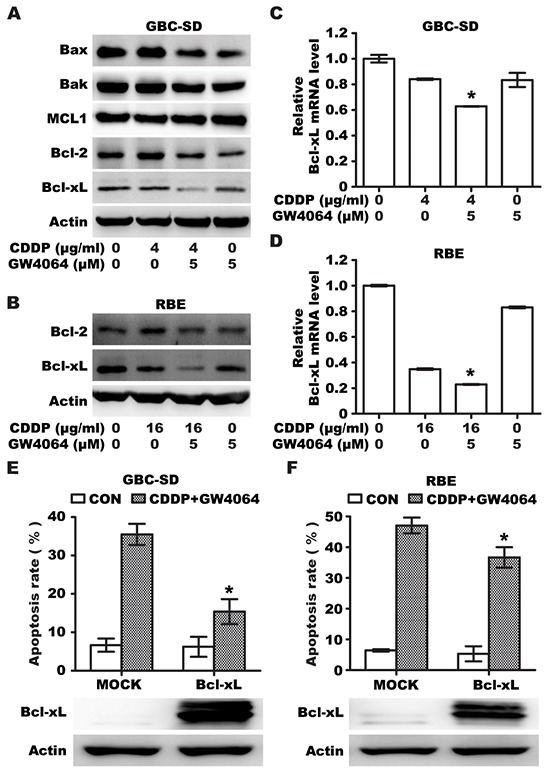
FXR agonist GW4064/CDDP co-treatment additively inhibits Bcl-xl expression **A.** Protein levels of Bax, Bak, Bcl-2, MCL1 and Bcl-xL in GBC-SD cells treated with CDDP alone, GW4064 alone and CDDP/GW4064 combination for 36h. **B.** Protein levels of Bcl-2 and Bcl-xL in RBE cells treated with CDDP alone, GW4064 alone and CDDP/GW4064 combination for 36h. **C, D.** The mRNA levels of Bcl-xL in GBC-SD (C) and RBE (D) cells treated with CDDP alone, GW4064 alone and CDDP/GW4064 combination for 24h. Columns, mean of three experiments; bars, SD. *P <0.05, combination treatment group compared with CDDP-alone group. **E.** Apoptosis rate analysis using Annexin V/PI flow cytometry in GBC-SD cells transfected with Bcl-xL plasmid for 24h before treatment with CDDP (4μg/ml)/GW4064 (5μM) combination for 48 h. Columns, mean of three experiments; bars, SD. *P <0.05, Bcl-xL/CDDP+GW4064 group compared with MOCK/CDDP+GW4064 group. **F.** Apoptosis rate analysis using Annexin V/PI flow cytometry in RBE cells transfected with Bcl-xL plasmid for 24h before treatment with CDDP (16μg/ml)/GW4064 (5μM) combination for 48 h. Columns, mean of three experiments; bars, SD. *P <0.05, Bcl-xL/CDDP+GW4064 group compared with MOCK/CDDP+GW4064 group.

In order to examine the mechanisms that might explain the increased susceptibility to the drug, expression of Bcl-2 family of proteins were examined. We first determined the effect of GW4064 and/or CDDP on the expression of pro-apoptotic protein Bax/Bak and anti-apoptotic protein MCL1/Bcl-2/Bcl-xL in GBC-SD cells, and found that an additive reduction in Bcl-xL was observed in GBC-SD and RBE cells treated with a combination of GW4064 and CDDP, compared to treatment with either GW4064 or CDDP alone (Figure 3A), whereas the expression of other Bcl-2 family proteins were not markedly affected (Figure 3A). Similar results were obtained with RBE cells (Figure 3B). Bcl-xL was also significantly decreased by CDCA/CDDP combination in GBC-SD and RBE cells (Supplementary Figures S1A). This indicated that Bcl-xL serves as an important common target of the combination therapy among these apoptosis-relative proteins. We also found that GW4064 or CDDP or a combination of these drugs decreases the transcriptional level of Bcl-xL (Figure 3C, 3D), indicating FXR agonist/CDDP co-treatment could additively repress the expression of Bcl-xL.

To determine whether Bcl-xL expression contributes to resistance against apoptosis induced by CDDP/GW4064 combination in GBC and RBE cells, cells were transfected with a plasmid encoding Bcl-xL, and treated with CDDP/GW4064 combination for 48 h. Results showed that exogenous overexpression of Bcl-xL could impede CDDP/GW4064 co-treatment induced apoptosis in GBC and RBE cells (Figure 3E, 3F). These data suggested that Bcl-xL expression reduced the sensitivity of BTC cells to drug-induced apoptosis, and FXR agonist facilitated cytotoxicity of CDDP through suppression of Bcl-xL expression.

### FXR agonist/CDDP co-treatment additively inhibits STAT3 phosphorylation

**Figure 4:**
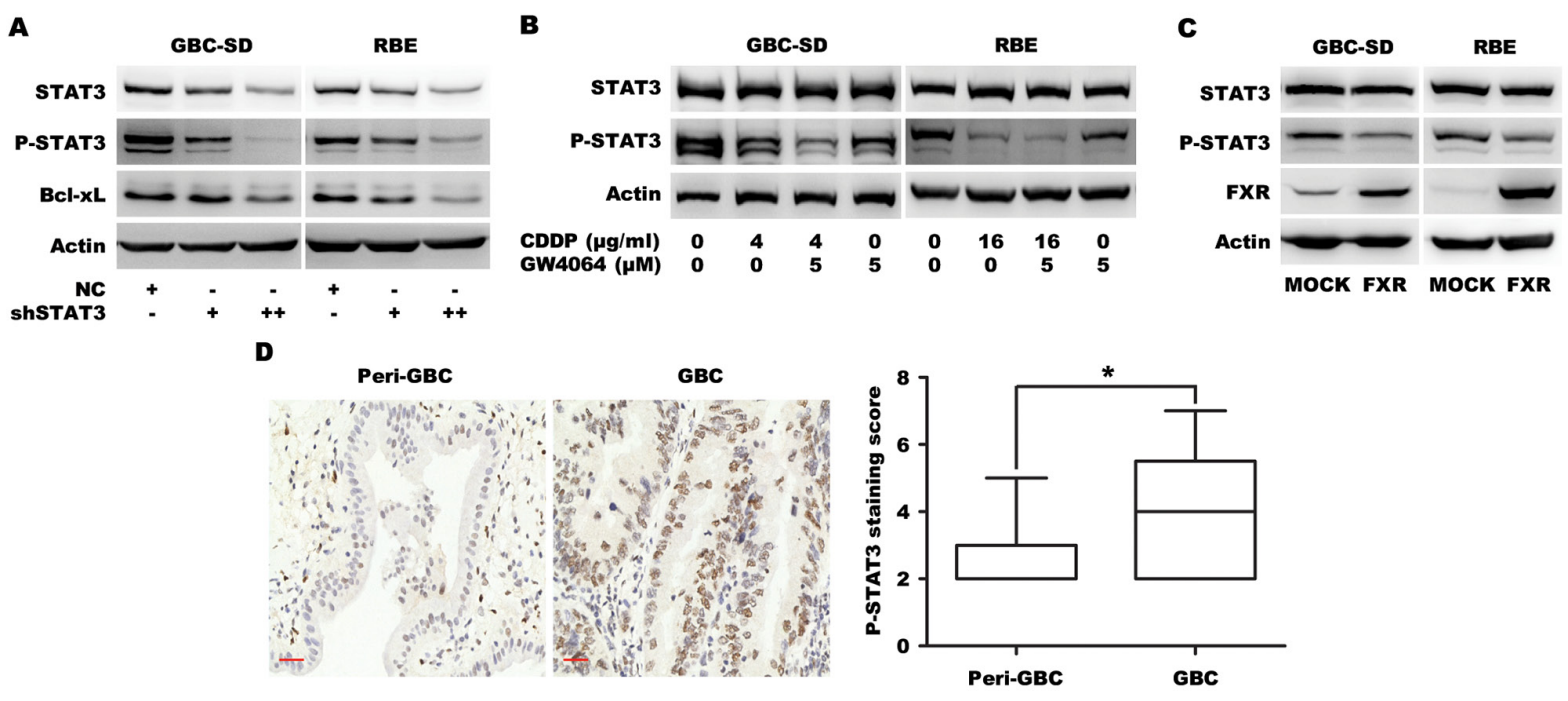
FXR agonist GW4064/CDDP co-treatment additively inhibits STAT3 phosphorylation **A.** Protein level of Bcl-xL in GBC-SD and RBE cells transfected with MOCK or shSTAT3 plasmid for 72 h. **B.** The STAT3 phosphorylation levels in GBC-SD and RBE cells that were treated with CDDP alone, GW4064 alone and CDDP/GW4064 combination for 24 h. **C.** Protein levels of FXR, P-STAT3 and STAT3 in GBC-SD and RBE cells transfected with MOCK or FXR plasmid for 72 h. **D.** The representative fields of patients' tissues of peri-gallbladder cancer (Peri-GBC, n=16) and gallbladder cancer (GBC, n=69) that were examined by P-STAT3 IHC. The nucleus brown staining represented positive signal for P-STAT3, and the cytoplasm was stained by hematoxylin. Scale bar=30 μm. The chart was the quantification of expression staining scores. *P <0.05, comparison between tumor and non-tumor tissues.

It has been shown that constitutive activation of STAT3 participates in oncogenesis through up-regulation of genes encoding apoptosis inhibitors including Bcl-xL [22]. GBC-SD and RBE cells were transfected with a plasmid encoding short hairpin RNA (shRNA) for STAT3 silencing. Knockdown of STAT3 significantly down-regulated phosphorylated STAT3 and Bcl-xL (Figure 4A), demonstrating STAT3 is responsible for Bcl-xL expression in BTC cells. GBC-SD and RBE cells were then treated with GW4064 and/or CDDP, which resulted in significant down-regulation of STAT3 phosphorylation by either GW4064 or CDDP and an additive effect by CDDP/FXR agonist co-treatment (Figure 4B). A similar additive effect on STAT3 phosphorylation was observed in GBC-SD and RBE cells treated with a combination of CDCA and CDDP, compared to treatment with either CDCA or CDDP alone (Supplementary Figures S1B). To further confirm the effect of FXR signaling on STAT3 phosphorylation in BTC cells, cells were transfected with an FXR cDNA plasmid. Exogenous overexpression of FXR induced significant dephosphorylation of STAT3 (Figure 4C). These data indicated that the synergistic effects of FXR agonist in BTC cells were due to the inhibition of STAT3 signaling.

Previous studies have shown that STAT3 is activated in human CC tissues [23, 24]. Therefore we determined the STAT3 phosphorylation in tissue microarray of human GBC using IHC and found that GBC tissues displayed higher level of STAT3 phosphorylation than non-tumorous tissues (Figure 4D), indicating that STAT3 is constitutively activated in GBC tissues. Collectively, these results suggest that STAT3 is constitutively activated in human BTC and is an important target to reverse chemotherapy resistance.

### FXR agonist could reverse CDDP-induced inhibition of SHP expression

**Figure 5:**
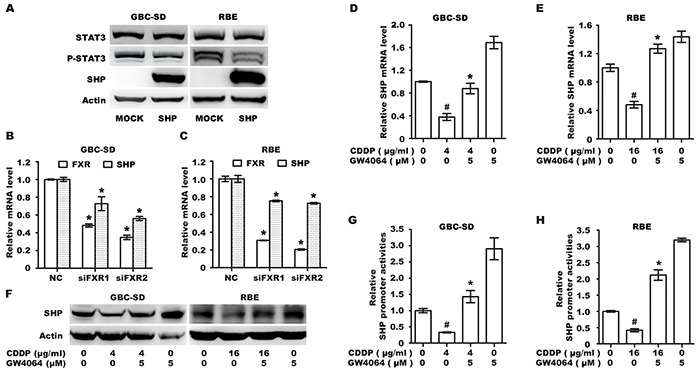
FXR agonist GW4064 could reverse CDDP-induced inhibition of SHP expression **A.** Protein levels of SHP, P-STAT3 and STAT3 in GBC-SD and RBE cells transfected with MOCK or SHP plasmid for 72 h. **B, C.** The mRNA levels of SHP in GBC-SD (B) and RBE (C) cells transfected with control siRNA (NC) or FXR siRNA (siFXR) for 72 h. Columns, mean of three experiments; bars, SD. *P <0.05, siFXR group compared with NC group. **D, E.** The mRNA level of SHP in GBC-SD (D) and RBE (E) cells treated with CDDP alone, GW4064 alone and CDDP/GW4064 co-treatment for 12 h. Columns, mean of three experiments; bars, SD. ^#^*P* <0.05, CDDP-alone group compared with control group. *P <0.05, combination treatment group compared with CDDP-alone group. **F.** The protein level of SHP in GBC-SD and RBE cells that were exposed to CDDP alone, GW4064 alone and CDDP/GW4064 co-treatment for 12 h. **G, H.** Luciferase reporter assay (RLA) in GBC-SD (G) and RBE (H) cells transfected with the SHP promoter reporter 24 h and then treated with CDDP alone, GW4064 alone and CDDP/GW4064 co-treatment for 12h. RLA was shown as mean±SD of three independent experiments. ^#^*P* <0.05, CDDP-alone group compared with control group. *P <0.05, combination treatment group compared with CDDP-alone group.

It has been shown that FXR functions as a transcription factor that regulates SHP expression [25], which in turn suppresses STAT3 activation [26]. We investigated whether the effect of CDDP/FXR agonist on STAT3 phosphorylation in BTC cells was due to regulation of SHP expression. First, cells were transfected with SHP cDNA plasmid to confirm the causative link between SHP expression and STAT3 phosphorylation in BTC cells. Results showed that overexpression of SHP induces significant dephosphorylation of STAT3 (Figure 5A). Next, GBC-SD and RBE cells were transfected with siRNA oligonucleotide for silencing FXR expression. Knockdown of FXR significantly suppressed the mRNA level of SHP (Figure 5B, 5C), suggesting that FXR is responsible for the transcription of SHP in BTC cells. Finally, we found CDDP alone significantly decreased both mRNA and protein levels of SHP, while CDDP/FXR agonist co-treatment resulted in an opposite effect (Figure 5D, 5E, 5F). Additionally, a reporter assay was conducted with phSHP-Luc construct. As shown in Figure 5G, 5H, treatment with the specific FXR agonist GW4064 significantly increased the basal transcriptional activity of SHP promoter and reversed CDDP-induced inhibition. These results suggest that up-regulation of SHP may be an important molecular mechanism underlying the additive inhibition of STAT3 phosphorylation by CDDP/FXR agonist co-treatment.

### FXR agonist markedly sensitizes the tumor xenografts to CDDP cytotoxicity without displaying obvious systemic toxicity in vivo

**Figure 6:**
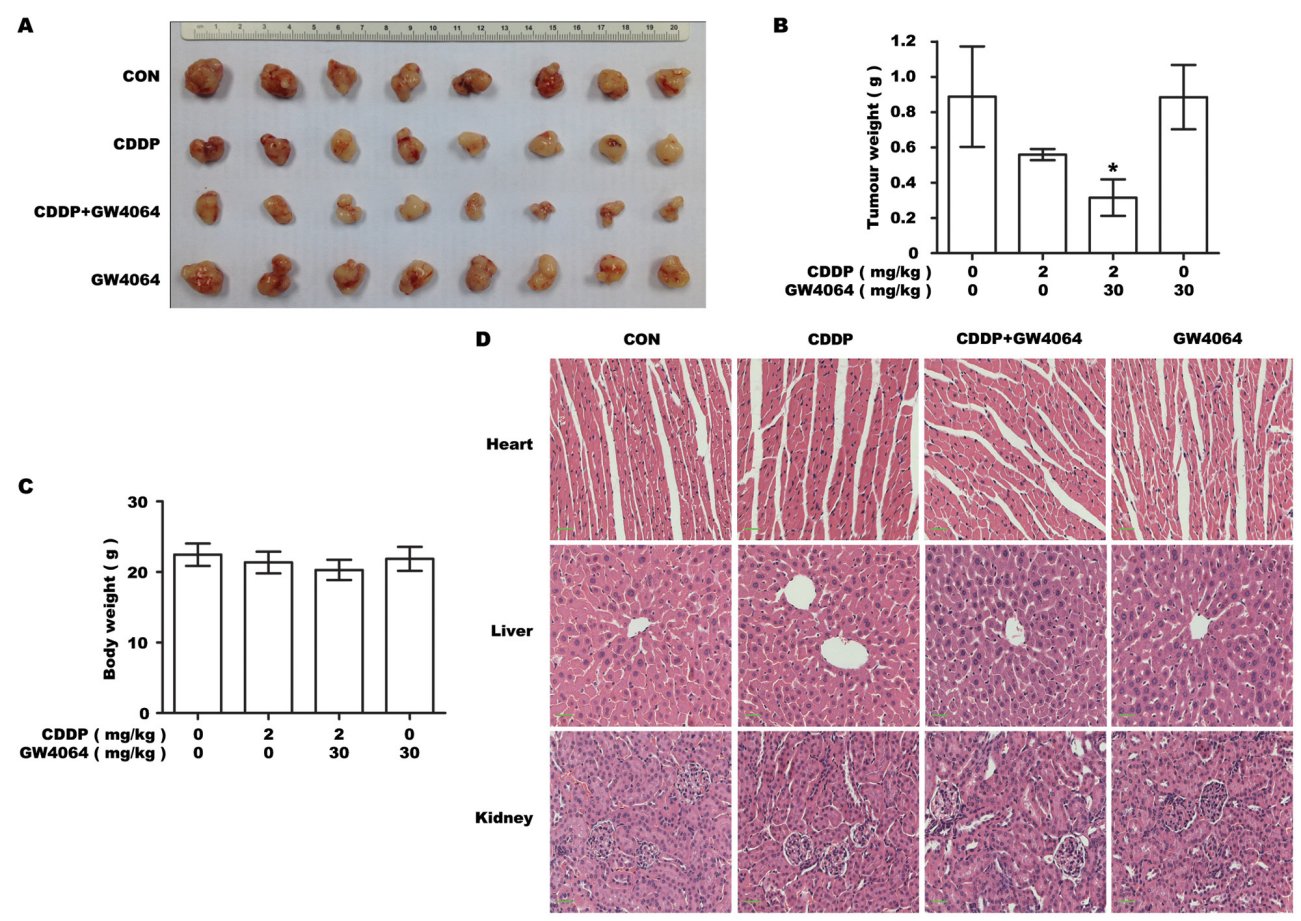
FXR agonist markedly sensitizes the tumor xenografts formed by GBC-SD cells to CDDP cytotoxicity without displaying obvious systemic toxicity in vivo The tumor-bearing mice were injected intraperitoneally with dissolvent, 30 mg/kg GW4064 alone, 2 mg/kg CDDP alone and CDDP/GW4064 coadministration (n=8/group). **A.** Photograph of transplanted tumors after the mice were exposed to treatments. **B.** Average weight of transplanted tumors after the mice were exposed to treatments. *P <0.05, combination treatment group compared with CDDP-alone group. **C.** Average body weight of tumor-bearing mice. **D.** Histology of livers, kidneys and hearts of tumor-bearing mice. Scale bar: 30μm.

Our in vitro experiments showed that drug-resistant phenotype of BTC cells could be partially overcome by using FXR agonist in combination with chemotherapeutic drug CDDP. To verify this effect in vivo, GBC-SD cells were transplanted into nude mice which were treated with GW4064 in combination with CDDP for 30 days. Our results showed that mice treated with the combined therapy had significantly smaller tumors than mice in other groups (Figure 6A, 6B). To evaluate the systemic toxic effects of the combined treatment, changes in bodyweight and any pathological changes in major organs were examined. No notable differences were observed between the groups (Figure 6C, 6D). These results demonstrate that FXR agonist/CDDP co-treatment had no obvious toxic effects on normal tissues in vivo.

**Figure 7:**
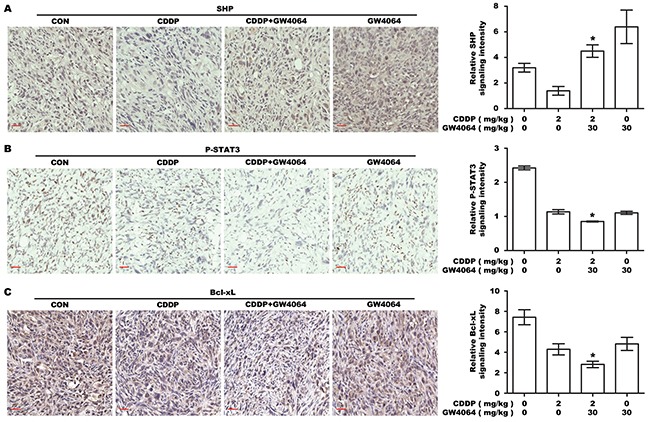
The effect of FXR agonist/CDDP co-treatment on SHP-STAT3-Bcl-xL signaling in vivo **A.** IHC staining for the expression of SHP in transplanted tumor tissues. The nucleus and cytoplasm brown staining represented positive labeling for SHP. Scale bar: 30 μm. The chart was the quantification of SHP in xenografted tumors. *P <0.05, combination treatment group compared with CDDP-alone group. **B.** IHC staining for P-STAT3. The nucleus brown staining represented positive signal for P-STAT3. Scale bar: 30 μm. The chart was the quantification. *P <0.05, combination treatment group compared with CDDP-alone group. **C.** IHC staining for Bcl-xL. The cytoplasm brown staining represented positive labeling for Bcl-xL, and the nucleus was stained by hematoxylin. Scale bar: 30 μm. The chart was the quantification. *P <0.05, combination treatment group compared with CDDP-alone group.

To examine the effect of FXR agonist/CDDP co-treatment on SHP-STAT3 signaling in vivo, IHC was performed on the tumor xenografts. As shown in Figure 7A, SHP expression in tumors was down-regulated by CDDP, but significantly reversed by GW4064/CDDP combined treatment. Phosphorylation of STAT3 and expression of Bcl-xL in tumors were down-regulated by CDDP, and in particular, more significantly by GW4064/CDDP combined treatment (Figure 7B, 7C). These results were in agreement with the results of the in vitro experiments.

## DISCUSSION

The “Toxic Bile” concept has been proposed to explain the effects of bile acids on cholestatic liver diseases [27]. Other than cholelithiasis, studies have also found that there is an excess risk of malignant tumors in some organs exposed to high concentration of bile acids, such as in the gastrointestinal tract [28]. FXR is an orphan nuclear receptor (NR) for bile acids, and is important in bile acid homeostasis, as well as in glucose and lipid metabolism [29]. Recent evidence further suggests a key role for FXR in apoptosis and cancer [30]. Previously, we have shown the inhibitory effect of FXR agonist GW4064 and CDCA on human BTC cell line QBC939 in vitro and in vivo [14, 16]. FXR agonists have been used in some other tumors, such as liver, colon, breast cancer [12, 13, 17, 31]. In the present study, we validated the above results in two additional drug-resistant BTC cell lines GBC-SD and RBE, and further demonstrated that FXR agonists can enhance cytotoxicity of CDDP. Interestingly and promisingly, our in vivo data shows that GW4064 can effectively enhance the anticancer effect of CDDP, with little systemic toxic effects. FXR agonist can prevent CDDP-induced kidney injury, the underlying mechanism of which may be associated with anti-fibrotic, anti-inflammatory, and anti-apoptotic effects through SHP induction [32]. These data provide strong evidence in favour of developing strategies aimed at reactivating FXR for treatment of BTC, either alone or combined with chemotherapy.

Our study further verified that apoptosis of BTC cells was additively induced by co-treatment of GW4064 with CDDP in a caspase-dependent manner, indicating that FXR agonist exerted synergistic anticancer actions via apoptotic pathways. The Bcl-2 protein family plays a central part in the control of apoptosis [33]. Bcl-2, Bcl-xL and Mcl-1 are anti-apoptotic members, whereas Bax, Bak and Bid are pro-apoptotic. During apoptosis, the permeability of the mitochondrial membrane increases, leading to a loss of membrane potential and release of cytochrome c into the cytosol. The Bcl-2 and Bcl-xL proteins bind to the outer membrane of the mitochondrion and prevent the release of cytochrome c. The pro-apoptotic members, such as Bax and Bak, are responsible for permeabilizing the membrane under stress and promoting the release of cytochrome c from the mitochondria. However, Kim et al. have reported that Bcl-xL makes a greater contribution to the sensitivity of BTC cells to parthenolide-induced apoptosis than other members of Bcl-2 family in BTC cells [34]. Similarly, our study demonstrates that GW4064/CDDP combination increases apoptosis due to significant down-regulation of Bcl-xL. Anti-apoptotic members of the Bcl-2 protein family (Bcl-2, Bcl-xL, and Mcl-1) had been previously investigated in human BTC tissues, and it was found that Bcl-xL is expressed frequently [35]. These results reveal that the reduced expression of Bcl-xL determines the increase of drug susceptibility of BTC cells.

As previous reports, SHP activates apoptosis by translocating to mitochondria, binding to the anti-apoptotic protein Bcl-2, and disrupting Bcl-2/Bid interaction to cause cytochrome c release [19]. In the present study, we found a new mechanism that activation of FXR-SHP pathway could significantly induce dephosphorylation of STAT3 and inhibit expression of its target Bcl-xL. Activated STAT signaling in human tumors provide novel molecular targets for therapeutic intervention [22, 36]. Previous studies have found that IL-6/STAT3 signaling is aberrant in human CC cells and CC tissues, with prolonged and sustained STAT-3 phosphorylation [23, 24]. In this study, prolonged and sustained STAT-3 phosphorylation was also found in human GBC tissues. Furthermore, we discovered that CDDP alone could inhibit STAT3 phosphorylation, and GW4064/CDDP co-treatment could additively induce dephosphorylation of STAT3 and inhibit expression of Bcl-xL. These data suggest that activating FXR could attenuate STAT3 activation, which might be the reason why FXR agonist could enhance cytotoxicity of CDDP in BTC cells.

Because of the anti-inflammatory actions, FXR has received much attention as a potential therapeutic target. In the animal model of LPS-induced liver injury, administration of the FXR natural ligand CDCA could attenuate hepatocyte inflammatory damage, reduce transaminase activities, suppress inflammation mediators (IL-6, TNF-α and ICAM-1) expression and inhibit STAT3 phosphorylation (activation) [37]. Isomoto et al. has also found similar results in cholangiocarcinoma [38]. SHP is an atypical member of NR superfamily because it lacks a DNA-binding domain but contains a putative ligand binding domain [39]. SHP executes its regulatory function through modes of protein-protein interaction. A recent study about hepatic insulin resistance has shown that SHP inhibited STAT3 activation through protein-protein interactions with STAT3 in mouse models [26]. We also confirmed that SHP inhibits STAT3 phosphorylation in human BTC cells. Many NRs and transcription factors have been reported to target the SHP promoter and stimulate SHP gene expression, including FXR [18]. Our study for the first time demonstrates that FXR plays an important role in stimulating SHP gene expression in BTC cells. To date, many studies have shown that the FXR ligand GW4064 [25, 40], androsterone [41], bile acids (BA) and CDCA [42, 43] are potent inducers of SHP expression. GW4064 is known as one of the most potent synthetic FXR agonists and has no activity on other nuclear receptors at concentrations up to 1 mM [44, 45]. In our study, CDDP and GW4064 were administered in GBC-SD and RBE cells, and it was discovered that CDDP could inactivate the transcription level of the SHP promoter and subsequently negatively regulate the expression of SHP at both mRNA level and protein level, which was reversed by GW4064. These data suggest that activating FXR-SHP pathway could attenuate the negative effect of CDDP, which results in an additional inhibition of STAT3 phosphorylation and a state more susceptible to the cytotoxic effects of chemotherapeutic agents in BTC cells. But why CDDP could inhibit STAT3 phosphorylation in BTC cells is unclear. This important mechanistic detail remains to be further investigated.

**Figure 8:**
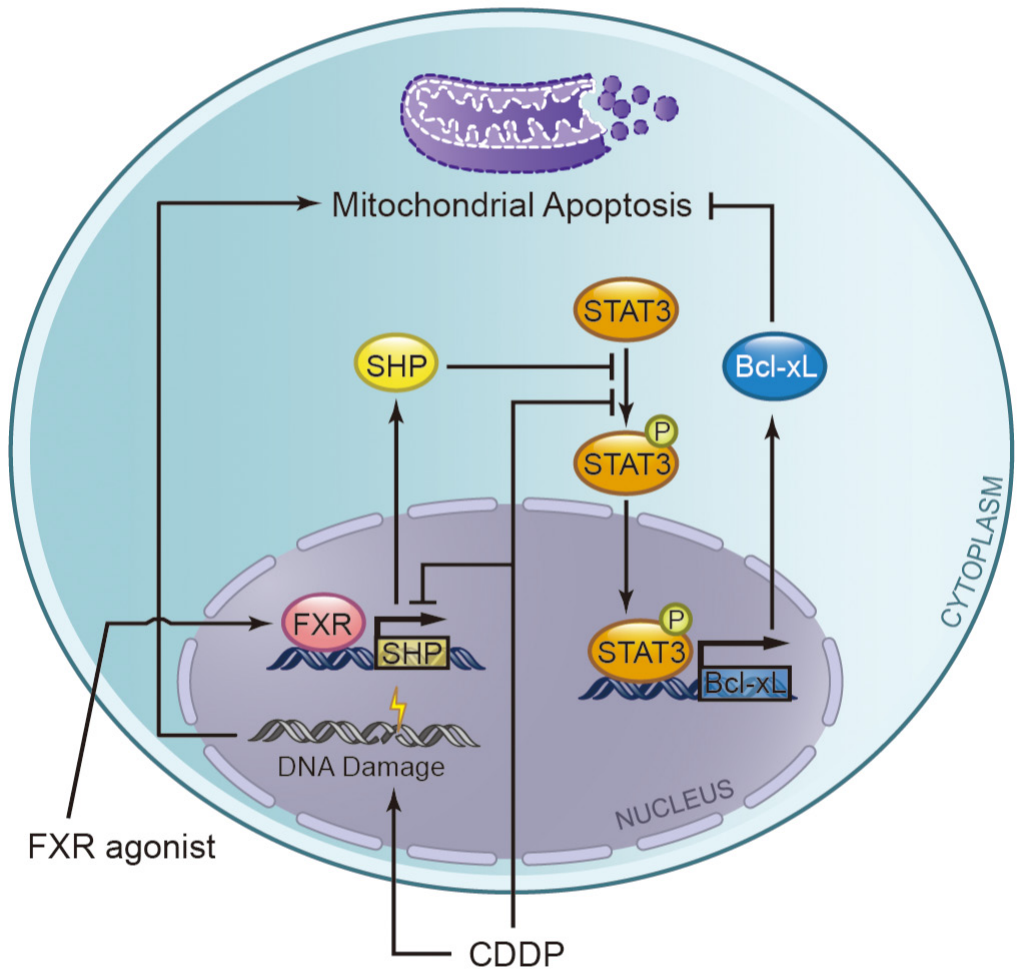
A hypothetical schematic representation of the effects of the combination of cisplatin (CDDP) plus FXR agonist on biliary tract cancer cells FXR agonists can bind to its receptor FXR and then activate the SHP expression, which overcome CDDP mediated downregulation of SHP, and subsequently stimulates apoptosis via blocking anti-apoptotic protein Bcl-xL. Mechanistically, SHP inhibits STAT3 phosphorylation and then represses the expression of Bcl-xL.

In conclusion, induction of FXR activity might increase the susceptibility of BTC cells to apoptosis following chemotherapy (Figure 8). Additionally, the natural FXR agonist CDCA has been used in clinic to improve bile excretory function. Various synthetic FXR agonists have emerged [46], and currently phase I clinical trials are undergoing to study the effectiveness of the synthetic FXR agonist apomine in treating patients who have advanced or metastatic solid tumors and have not responded to previous treatments [National Institutes of Health (NIH), Clinical Trials. Available at http://www.clinicaltrials.gov]. Therefore, targeting FXR and improving its function might be a promising strategy for the treatment of BTC.

## MATERIALS AND METHODS

### Cells and reagents

The human BTC cell line GBC-SD and RBE were obtained from the Type Culture Collection of the Chinese Academy of Sciences (Shanghai, China) in 2012. SGC996 and QGC939 cells were obtained from Shanghai Tongji University (Shanghai, China) in 2007. These cell lines were characterized using morphological assays, cytogenetic analysis and short tandem repeating sequence PCR (STR-PCR) when these cells were released by the cell banks. We further examined these cells by morphological assays and tumorigenesis in nude mice in our previous and current studies [14, 16, 47]. All cell lines were maintained in DMEM medium (GibcoBRL, Gaitherburg, MD, USA). These media were supplemented with antibiotics and 10% fetal bovine serum. Cells were cultured in a humidified atmosphere with 5% CO_2_ at 37°C. CDDP were obtained from Qilu Pharmaceutical Co., Ltd. (Ji Nan, China). GW4064 and chenodeoxycholic acid (CDCA) were purchased from Sigma (St. Louis, MO, USA).

### Cell viability assay

Cells were seeded at 1.5×10^4^ cells per well in 96-microculture-well plates. After exposed to the agents as indicated for 48 h, cell viability was assayed using the 3-(4,5-dimethylthiazol-2-yl)-2,5-diphenyl-tetrazolium bromide (MTT) (Sigma, St. Louis, MO, USA).

### Cell apoptosis analysis

Cells were treated with drugs and then apoptotic rates were assessed with flow cytometry using Annexin V-fluorescein isothiocyarate (Annexin V-FITC)/propidium iodide (PI) kit (BD Pharmingen, San Diego, CA, USA). Samples were prepared according to the manufacturer's instruction and analyzed by flow cytometry on FACS Calibur (Becton Dickson, San Diego, CA, USA).

### Immunoblotting (IB)

The whole-cell extracts were prepared, and the protein concentrations were determined by BCA assay. The extracts were separated by sodium dodecyl sulfate-polyacrylamide gel electrophoresis (SDS-PAGE) and transferred to polyvinylidene fluoride (PVDF) membrane. The membrane was incubated with primary antibodies and peroxidase-conjugated secondary antibodies and then visualized using an enhanced chemiluminescence system kit (Pierce, Rockford IL, USA). The antibodies against caspase 3 (Cell Signaling Technology, Beverly, MA, #9665), cleaved caspase 3 (Cell Signaling Technology, Beverly, MA, #9664), Bax (Cell Signaling Technology, Beverly, MA, #2772), Bak (Santa Cruz Biotechnology, Santa Cruz, CA, sc-832), MCL1 (ABcam, UK, ab32087), Bcl-2 (Cell Signaling Technology, Beverly, MA, #2870), Bcl-xL (ABcam, UK, ab32370), FXR (ABcam, UK, ab126602), STAT3 (Cell Signaling Technology, Beverly, MA, #4904), P-STAT3 (Cell Signaling Technology, Beverly, MA, #9145), SHP (Santa Cruz Biotechnology, Santa Cruz, CA, sc-271511) and β-actin (ABcam, UK, ab6276) were used.

### Real-time PCR

Total RNA was isolated from cell lines using TRIZOL (Invitrogen, Carlsbad, CA, USA) according to the manufacturer's instructions. Two microgram of the isolated total RNA was reverse-transcribed using random primers and AMV reverse transcriptase (Promega, Madison, WI, USA) for 5min at 70°C, 5min on ice and 60 min at 37°C. Quantitative real-time PCR was performed on the ABI Prism 7500 system (Applied Biosystems, Foster City, CA) using SYBR Green and following the manufacturer's instructions. The primers for FXR were 5′-GATGCCTGTAACAAAGAAGCCCC-3′ and 5′-CACACAGTTGCCCCCGTTTTTAC-3′. The primers for SHP were 5′-TTAACCCCGATGTGCCAGG-3′ and 5′-GGTCGGAATGGACTTGAGGG-3′. The primers for Bcl-xL were 5′-TGCAGGTATTGGTGAGTCGG-3′ and 5′-AAGCGTTCCTGGCCCTTTC-3′. The primers for GAPDH were 5′-GAAGGTGAAGGTCGGAGTC-3′ and 5′-GAAGATGGTGATGGGATTTC-3′.

### Tissue microarray (TMA)

The tissue microarray of human gallbladder cancer was purchased from Shanghai Outdo Biotech Co., Ltd. (Shanghai, China). There was a cohort of 69 cases enrolled in the study. TMA slides were deparaffinised in xylene and rehydrated through a ladder of graded ethanol (absolute ethanol, 90%, 80%, 70% and distilled water). Antigen retrieval was done in citric acid antigen repair solution (pH 6.0) in an autoclave for 5 minutes (100°C) before being processed in an automatic immunohistochemistry staining machine according to standard procedures (Autostainer, Dako, Sweden). All antibodies were applied for 30 minutes at room temperature. The primary antibody P-STAT3 was used at 1:50 dilution. Immunostainings were detected via DAKO Cytomation envision/HRP kit K5007. The primary antibody was omitted from negative controls. Immunostainings were scored by blinded observers according to intensity and percentage of positive cells. Staining intensity was scored as 0 (negative), 1 (weak), 2 (moderate), and 3 (strong). The percentage of positive cells was scored as 0 (0%), 1 (1-25%), 2 (26-50%), 3 (51-75%), and 4 (76-100%). The sum of the intensity and percentage scores was used as the final score (0-7). Tumors with a final staining score of >3 were considered to high expression or else low expression.

### Luciferase reporter assay

The luciferase reporter plasmid was constructed as described previously [48]. The promoter region (−572 to +10) of human SHP was amplified by PCR and then inserted into the luciferase reporter plasmid pGL3-Basic (Promega, Madison, WI, USA), and the resulting vector was named as phSHP-Luc. GBC-SD cells or RBE cells were grown to 70% to 80% confluence in 24-well plates. Cells were transiently cotransfected with a luciferase reporter (phSHP-Luc) (0.2 μg per well) and Renilla (0.02 μg per well) using the Lipofectamine 2000 reagent (Invitrogen, Carlsbad, CA, USA) according to the manufacturer's instructions. At 24 h post-transfection cells were incubated with GW4064 for 12 h. The expression levels of luciferase and Renilla in the cell lysates were monitored in a FB12 luminometer (Berthold, Germany) after the substrates (Promega, Madison, WI, USA) were added. Relative luciferase activity (RLA) was obtained by normalizing the luciferase activity with the Renilla activity.

### Cell transfection

FXR cDNA plasmid was kindly provided by Susan P.C. Cole, Queen's University at Kingston, Canada. Bcl-xL cDNA plasmid was purchased from Sino Biological Inc. (Beijing, China). Other plasmids were obtained from our laboratory. FXR siRNA was synthesized by RiboBio Co., Ltd. (Guangzhou, China). cDNA plasmid (Bcl-xL, FXR, SHP), shRNA plasmid (STAT3) or FXR siRNA was transiently transfected, using the Lipofectamine 2000 reagent (Invitrogen, Carlsbad, CA, USA) according to the manufacturer's instructions. A nonsense plasmid or control siRNA were also transfected as mock. Then cells were exposed to an additional treatment as indicated.

### In vivo study in tumor-bearing mice

All animal experiments were done in accordance with institutional guidelines for animal welfare. GBC-SD cells were harvested, washed, and resuspended in serum-free optimum medium and then injected subcutaneously into 5-week old BALB/c-nu/nu mice, with 6×10^6^ cells per mice (n = 8 mice per group, purchased from Shanghai Experimental Animal Center, Shanghai, China). Tumor size was measured with a caliper, and tumor volumes were calculated using the formula [49]: V=Π/6×length×width^2^. When the tumor size was approximately 50 mm^3^, mice were sorted into 4 equal groups. Then the tumor-bearing mice were intraperitoneally administered with dissolvent, GW4064 (30 mg/kg), CDDP (2mg/kg), GW4064/CDDP every other day. The mice were sacrificed after 30 days, and body weight and tumor weight were measured. Hearts, livers and kidneys of the mice were histologically examined to determine the systemic toxicity. The tumor xenografts were collected and their paraffin-embedded sections were subjected to IHC (immunohistochemistry) as previously described [50]. The antibodies against SHP (1:50; Santa Cruz Biotechnology, Santa Cruz, CA, sc-271511), P-STAT3 (1:50; Cell Signaling Technology, Beverly, MA, #9145) and Bcl-xL (1:50; ABcam, UK, ab32370) were used. Quantitative image analysis for the areas of immunostaining was conducted using Zeiss KS400 software.

### Statistical analysis

Data were shown as mean values ± S.D. ANOVA (analysis of variance) was applied for comparison of the means of two or multiple groups, in which SNK (Student-Newman-Kewls) was further used for comparison of each two group. SPSS 11.0 software (SPSS, Inc., Chicago, IL) was used for statistical analysis. A value of *P* <0.05 was considered significant.

## SUPPLEMENTARY FIGURE


