# Supplementary material for: FXR agonists enhance the sensitivity of biliary tract cancer cells to cisplatin via SHP dependent inhibition of Bcl-xL expression
Source: Oncotarget. 2016 Apr 25;7(23):34617–29. doi: 10.18632/oncotarget.8964 (PMC5085180; doi:10.18632/oncotarget.8964)
Supplement: Supplementary file 1 [file oncotarget-07-34617-s001.pdf]

SUPPLEMENTARY FIGURE

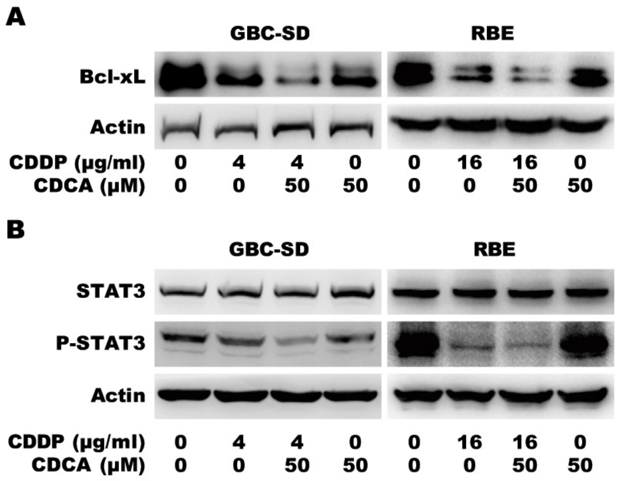

Supplementary Figure S1: FXR agonist CDCA/CDDP co-treatment additively inhibits STAT3-Bcl-xL signaling. A. Protein levels of Bcl-xL in GBC-SD and RBE cells treated with CDDP alone, CDCA alone and CDDP/CDCA combination for 36h. **B.** The STAT3 phosphorylation levels in GBC-SD and RBE cells treated with CDDP alone, CDCA alone and CDDP/CDCA combination for 24 h.
